# Constant low-to-moderate mechanical asymmetries during 800-m track running

**DOI:** 10.3389/fspor.2024.1278454

**Published:** 2024-01-19

**Authors:** Olivier Girard, Grégoire P. Millet, Jean-Paul Micallef

**Affiliations:** ^1^ Exercise and Sport Science Department, School of Human Sciences, The University of Western Australia, Perth, WA, Australia; ^2^ Institute of Sport Sciences, ISSUL, University of Lausanne, Lausanne, Switzerland; ^3^ Movement to Health (M2H), Montpellier-1 University, EuroMov, Montpellier, France

**Keywords:** symmetry angle scores, fatigue, running mechanics, middle-distance running, ground reaction forces

## Abstract

**Introduction:**

Modifications in asymmetry in response to self-paced efforts have not been thoroughly documented, particularly regarding horizontally-derived ground reaction force variables. We determined the magnitude and range of gait asymmetries during 800 m track running.

**Methods:**

Eighteen physical education students completed an 800 m self-paced run on a 200 m indoor track. During the run, vertical and horizontal ground reaction forces were measured at a sampling frequency of 500 Hz using a 5 m-long force platform system, with data collected once per lap. The following mechanical variables were determined for two consecutive steps: contact time and duration of braking/push-off phases along with vertical/braking/push-off peak forces and impulses. The group mean asymmetry scores were evaluated using the “symmetry angle” (SA) formula, where scores of 0% and 100% correspond to perfect symmetry and perfect asymmetry, respectively.

**Results:**

There was no influence of distance interval on SA scores for any of the nine biomechanical variables (*P* ≥ 0.095). The SA scores were ∼1%–2% for contact time (1.3 ± 0.5%), peak vertical forces (1.8 ± 0.9%), and vertical impulse (1.7 ± 1.0%). The SA scores were ∼3%–8% for duration of braking (3.6 ± 1.1%) and push-off (3.2 ± 1.4%) phases, peak braking (5.0 ± 2.1%) and push-off (6.9 ± 3.1%) forces as well as braking (7.6 ± 2.3%) and push-off (7.7 ± 3.3%) impulses. The running velocity progressively decreased at 300 m and 500 m compared to that at 100 m but levelled off at 700 m (*P* < 0.001).

**Discussion:**

There were no modifications in gait asymmetries, as measured at 200-m distance intervals during 800-m track running in physical education students. The 800 m self-paced run did not impose greater mechanical constraints on one side of the body. Experimental procedures for characterizing the gait pattern during 800 m track running could be simplified by collecting leg mechanical data from only one side.

## Introduction

Middle-distance events (i.e., 800 m and 1,500 m) are part of the programme contested at major athletics championships. These races are associated with large cardio-metabolic demands, as shown by attainment of maximal oxygen uptake only ∼45 s after the onset of an overground 800 m run (1). In contrast to physiological requirements which are well established, the biomechanical aspects of the 800 m race have been less studied, yet available evidence indicates that high mechanical constraints are placed on the neuromuscular system (2). For example, fatigued runners generated reduced peak braking and push-off forces, resulting in a shorter stride length during 800 m track running (3). Collectively, these observations suggest that as runners become fatigued, they become less capable of tolerating ground impact.

One important methodological limitation of previous analyses is that minimal asymmetry between legs is often assumed. This assumption is often made to simplify data collection, such as unilateral data collection due to camera positioning constraints or a limited number of available force plates (4), and/or data analysis, which involves pooling of data from both limbs (5). This is not in keeping with the substantial body of knowledge suggesting that most uninjured runners present low-to-moderate gait asymmetries (6, 7). This suggests that collecting unilateral data may not be the optimal approach for accurately describing the biomechanical effects of fatigue during middle-distance events. This is because there is a possibility that the lower extremities fatigue at different rates (8).

Several experiments have documented asymmetries in fatigued runners. For example, Gao et al. (9) indicated that a fatigue protocol induced by running resulted in increased asymmetry in knee flexion angle, hip flexion angle, hip extension angle, and the hip flexion moment. Furthermore, amateur runners completing a fixed-pace 10 km treadmill run (∼45 min) exhibited increased asymmetry. The asymmetric patterns were primarily observed in the vertical oscillation of the center of mass, as evidenced by 3D accelerometer data. Notably, significant changes occurred after approximately 15 min of running (10). Contrastingly, no noticeable differences in gait asymmetries were observed during repeated treadmill sprints in elite female Rugby Sevens players (11). Nevertheless, the current body of evidence regarding how fatigue affects asymmetries during running bouts is inconclusive, as highlighted in a systematic review conducted by Heil et al. (8).

In most previous asymmetry studies, experimentally-imposed exercise intensities (i.e., fixed running velocity) were prescribed to determine the effects of fatigue, with participants running for a certain distance [i.e., 10,000 m (7)] or duration [i.e., 130 min (12)] but also until exhaustion [i.e., at their maximal aerobic velocity (13)]. This approach likely results in an unnatural control of stride mechanical pattern, and is not representative of real-world scenarios where runners tend to modulate their pace tactically (14). While instrumented treadmills have mainly been used to quantify asymmetry for multiple steps (10, 15), treadmill running mechanics may differ from overground running during the stance phase [i.e., lower knee flexion and shorter contact times (16)]. When comparing maximal effort bend sprinting to straight-line sprinting, research unveils substantial differences between the left and right steps in mechanical variables, including ground contact time, touchdown distance, and hip flexion/extension and abduction/adduction angles (17). It is possible (although currently unknown) that the distinct functional roles of the left and right steps during bend running could have implications for the straight sections of an indoor track. To overcome this problem, modifications in asymmetry in response to self-paced efforts should be assessed during track running to reflect the actual running demands.

As reviewed by Heil et al. (8), most previous fatigue experiments focussed on assessing changes in asymmetries by examining potential pre-post differences (9, 18, 19), yet few have documented adjustments occurring at regular time points during the exhaustive protocol. Because asymmetry scores are largely metric-dependent, not only one parameter [e.g., contact time during a 5,000 m run (20)] should be measured to consider a runner as asymmetric (7). Another literature drawback is the use of separate (non-specific) tasks/procedures such as unilateral jump tests to detect asymmetry under fatigue (21), which may not depict “real” running demands since muscles are stressed differently. Finally, asymmetry assessments of exhaustive runs have often been limited to vertical ground reaction force (GRF) variables (7, 22). In the horizontal direction, whether braking and push-off are characterized by similar magnitude for bilateral leg differences, and if the onset and progression of fatigue-related asymmetries are also metric-dependent (i.e., phase duration, peak force, impulse), is largely undetermined.

The purpose of this study was to assess stride mechanical asymmetries, including phase duration, peak forces, and impulses, during an 800-m self-paced run conducted on an overground track. We hypothesized that variations in running mechanics between limbs would vary depending on the specific variable measured (greater for horizontally- vs. vertically-derived variables) and that these differences would become more pronounced as the distance covered increased.

## Methods

### Participants

A convenience sample of 18 male participants (mean age: 21.2 ± 2.8 years; mean height: 1.78 ± 0.40 m; mean body mass: 70.4 ± 6.6 kg) was recruited for this study. All participants were physical education students who had engaged in physical activities, including high-intensity efforts such as soccer and rugby, in the six months preceding the study. Although they were not specialists in middle-distance running, all participants were accustomed to running an 800 m distance three times a year as part of their physical performance assessment. Selection criteria included a history of training volume exceeding 3 h/wk on average (4.9 ± 1.2 h/wk), while a medical questionnaire was administered to exclude individuals with any lower limb injuries within the past three months. Participants provided written informed consent to participate in this study after receiving information about the procedures approved by the local ethical committee and in compliance with the Declaration of Helsinki.

### Protocol overview

While this study was conducted as part of a larger project investigating alterations in running mechanics during 800 m self-paced running (3), it is important to note that the primary outcome measures in the current study (gait asymmetries) do not overlap with previous analyses.

Participants commenced with a standardized warm-up comprising ten min of running at a speed of 10 km/h, followed by ten min of athletic drills, stretching, and three accelerated runs (interspersed with 1 min of passive rest) over a 50 m distance. The running velocity for these accelerated runs was chosen subjectively and corresponded to the start pace for an 800 m race, as described previously (23). After a 3 min recovery period, participants completed an 800 m time trial on a 200 m indoor Tartan track with banked corner. Each athlete ran individually and had the freedom to select their own pace to complete the 800 m time trial as quickly as possible. Split times at every 200 m were provided to the athletes, and verbal encouragement was given during the time trial. All tests were conducted at the same time of day, between 4 and 8 pm, with a consistent air temperature of 20–22°C.

### Data collection

At intervals equivalent to one lap (200 m), the vertical and anterior-posterior components of the GRF were assessed using a 5 m-long force platform system with a natural frequency of 200 Hz, as positioned at the end of a straight line (i.e., 100, 300, 500, and 700 m from the start line) (23). This system comprised five individual force plates, each measuring 1.00 m by 1.00 m, arranged in series and covered with a Tartan mat. The force plates were levelled with the stadium track. Kistler piezoelectric sensors (KI 9067, Kistler, Winterthur, Switzerland) were installed on each force platform.

Before conducting each test, the GRF signals were calibrated following the manufacturer's recommended procedure, with a sampling frequency of 500 Hz, using MP100 hardware (Biopac Systems Inc., Santa Barbara, CA, USA). The data were then stored for subsequent analysis using commercially available software (Acqknowledge 3.6.7, Biopac Systems Inc., CA, USA) (3).

The participants’ instantaneous running velocity on the force platform system was recorded using a radar Stalker ATS System™ (Radar Sales, Minneapolis, MN, USA) with a sampling frequency of 35 Hz. This radar device was positioned on a tripod at a height of 1 m, which approximately aligned with the height of the participants’ center of mass.

### Data analyses

During each lap, specifically at distance intervals of 100, 300, 500, and 700 m, there were typically two to four ground contacts registered on the force platform system. The number of contacts depended on factors such as step length and the distance between the first foot contact and the entry point of the force plate area. For each ground contact, the amplitude of the active peak of the vertical GRFs was measured. Data from the two highest consecutive steps, representing one stride within each 200 m interval, were selected for the final analysis.

### Running mechanical parameters

Contact and flight times (in seconds) were defined based on the vertical GRFs being either greater than or less than 10 N, following the method used by Girard et al. (3). Peak amplitudes of the vertical GRFs, as well as the peak braking and push-off forces, were calculated. The duration of the braking and push-off phases (in seconds) during ground contact was assessed using the anterior-posterior GRFs. Specifically, the braking phase was identified when the anterior-posterior GRF signal was negative, and the push-off phase when it was positive. Values for vertical, braking, and push-off impulses (in N·s^−1^) were computed by multiplying the effective force applied to the running surface by the corresponding foot-ground contact times for each of these phases.

### Symmetry angle

To assess inter-leg symmetry for each participant, the symmetry angle (SA) equation, as described by Zifchock et al. (24), was employed:

Symmetry angle (SA) =

$$\frac{\left|45^{\circ }-\left(\tan^{-1}\left[\frac{\mathrm{left}}{\mathrm{right}}\right]\right)\right|}{90^{\circ }}\times 100$$

but if

$$\left(45^{\circ }-\tan^{-1}\left[\frac{\mathrm{left}}{\mathrm{right}}\right]\right)>90^{\circ }$$

Then

$$\frac{\left|45^{\circ }-\left(\tan^{-1}\left[\frac{\mathrm{left}}{\mathrm{right}}\right]-180^{\circ }\right)\right|}{90^{\circ }}\times 100$$

The SA is calculated as the arctan function of the ratio between two values from each leg, with a SA score of 0% signifying perfect symmetry and 100% indicating perfect asymmetry.

### Statistical analysis

The data is presented as mean ± SD along with a 95% confidence interval (CI_95%_). To analyze the impact of the distance interval on the nine biomechanical variables, a repeated measures single-factor ANOVA was conducted across each 200 m interval (100, 300, 500, and 700 m). Mauchly's test of sphericity was employed to assess assumptions of variance across all ANOVA results. In the event of a significant main effect, a Bonferroni post-hoc multiple comparison was carried out. For each ANOVA, partial eta-squared (*η*²) was computed as a measure of effect size, with values of 0.01, 0.06, and above 0.14 considered as *small*, *medium*, and *large*, respectively (25). All statistical analyses were performed using SPSS statistical software version 27.0 (IBM Corp., Armonk, NY, USA), and the significance level was set at *P* < 0.05.

## Results

### Symmetry angle scores

**Figure 1:**
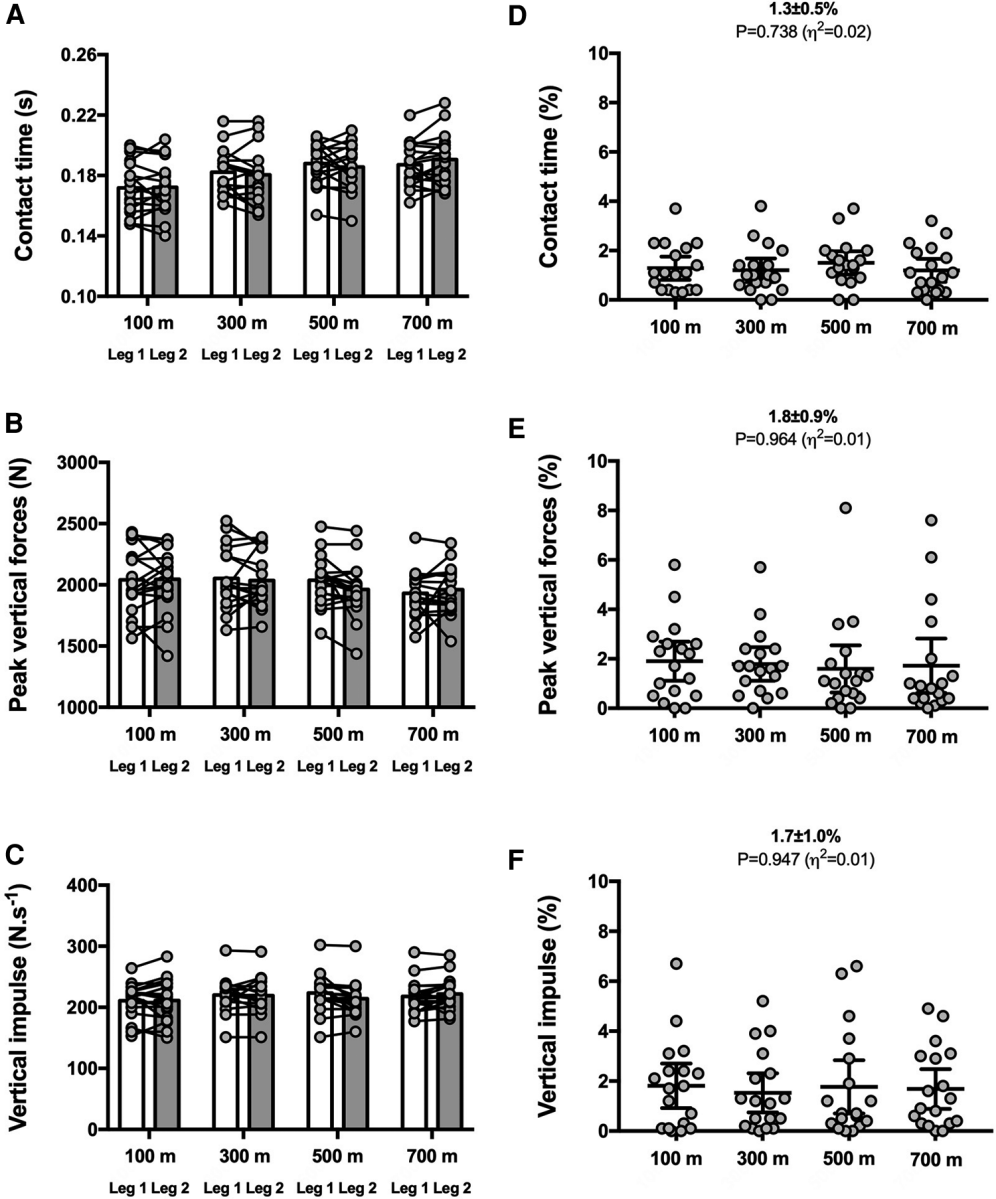
Vertical force-related variables for both legs (left panels) and symmetry angle scores (right panels) during the 800 m run. Contact time (**A**,**D**), peak vertical forces (**B**,**E**) and vertical impulse (**C**,**F**). Values are mean with 95% confidence interval (*n* = 18).

**Figure 2:**
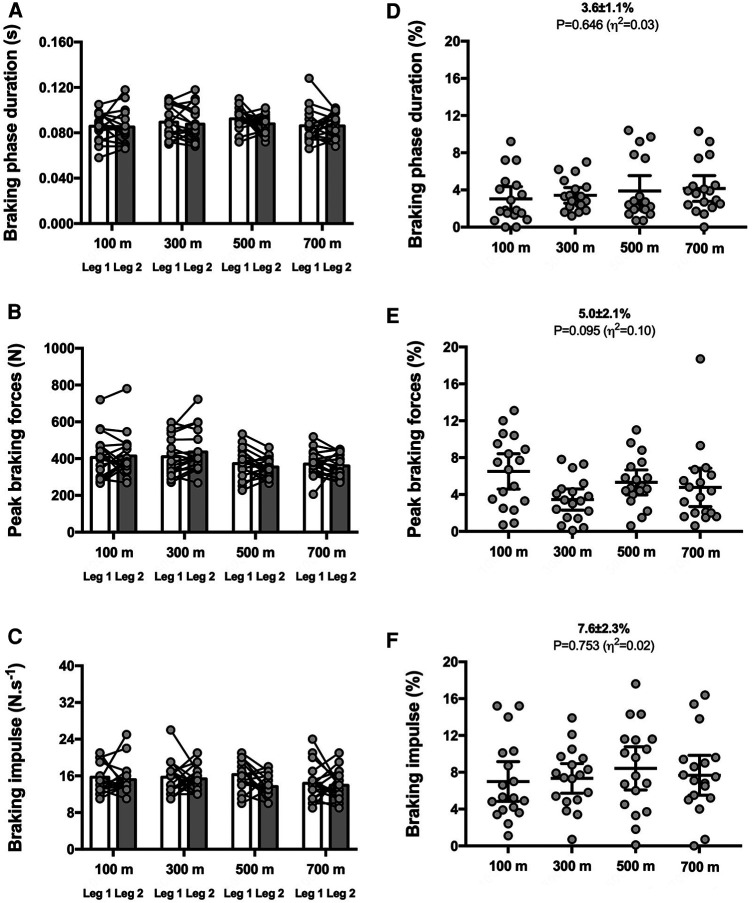
Braking phase-related variables for both legs (left panels) and symmetry angle scores (right panels) during the 800 m run. Braking phase duration (**A**,**D**), peak braking forces (**B**,**E**) and braking impulse (**C**,**F**). Values are mean with 95% confidence interval (*n* = 18).

**Figure 3:**
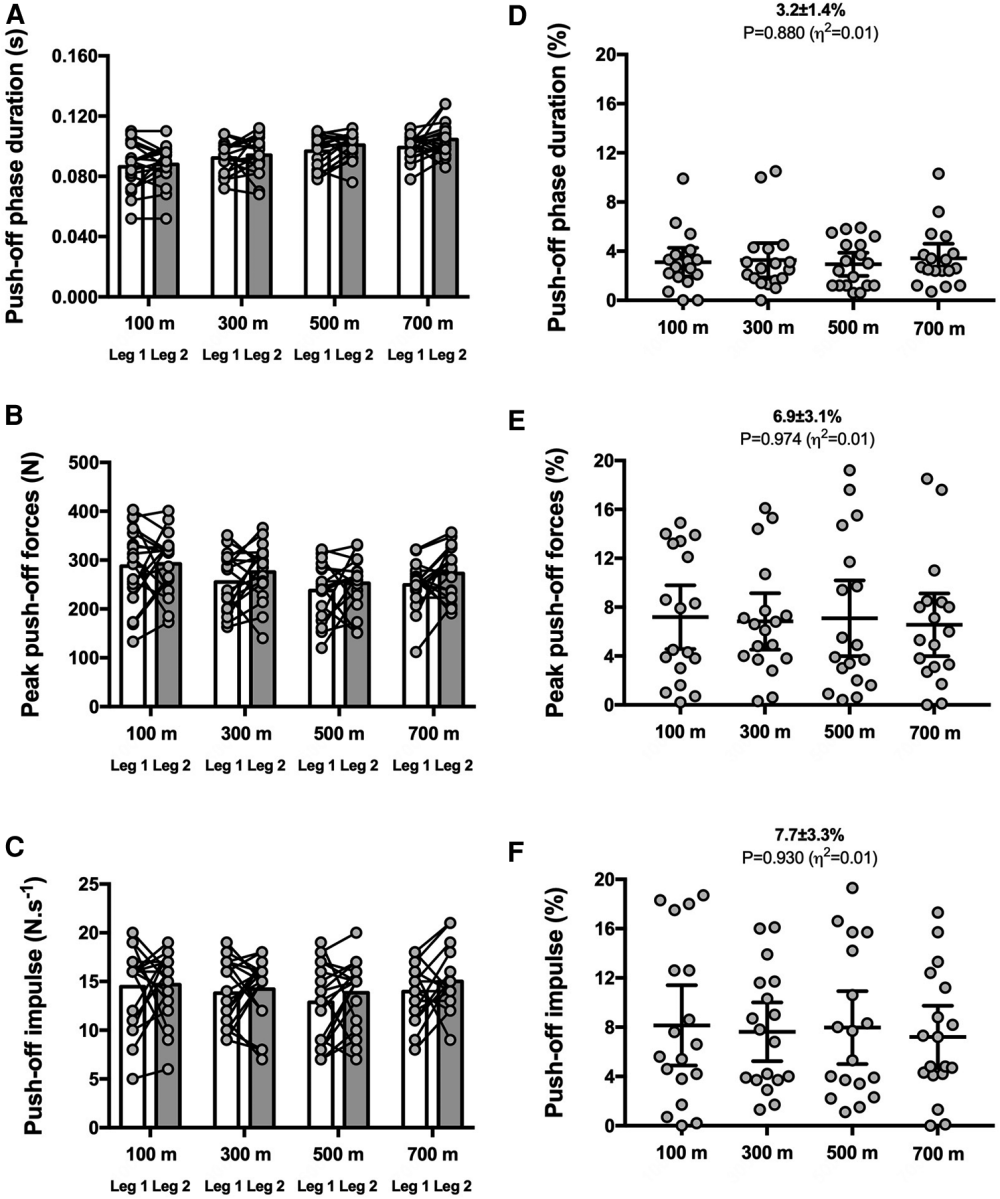
Push-off phase-related variables for both legs (left panels) and symmetry angle scores (right panels) during the 800 m run. Push-off phase duration (**A**,**D**), peak push-off forces (**B**,**E**) and push-off impulse (**C**,**F**). Values are mean with 95% confidence interval (*n* = 18).

The distance interval had no impact on SA scores for any of the nine biomechanical variables (*P* ≥ 0.095; see Figures 1–3). Therefore, the group mean and the range of SA scores are subsequently presented in the text as pooled values, which represent the average across the four distance intervals. This approach provides a meaningful benchmark for the expected magnitudes of asymmetry during an 800 m time trial for each specific metric.

The SA scores were ∼1%–2% for contact time [1.3 ± 0.5% (CI_95%_ 1.0–1.5); range: 0.6–2.2], peak vertical forces [1.8 ± 0.9% (CI_95%_ 1.3–2.2); range: 0.5–3.3], and vertical impulse [1.7 ± 1.0% (CI_95%_ 1.2–2.2); range: 0.1–3.8] (Figure 1).

The SA scores were ∼3%–8% for duration of braking [3.6 ± 1.1% (CI_95%_ 3.1–4.2); range: 1.7–5.3] and push-off [3.2 ± 1.4% (CI_95%_ 2.5–3.9); range: 1.4–5.9] phases, peak braking [5.0 ± 2.1% (CI_95%_ 4.0–6.1); range: 0.6–9.4] and push-off [6.9 ± 3.1% (CI_95%_ 5.4–8.5); range: 1.7–12.8] forces as well as braking [7.6 ± 2.3% (CI_95%_ 6.5–8.7); range: 2.7–12.4] and push-off [7.7 ± 3.3% (CI_95%_ 6.2–9.4); range: 3.2–13.7] impulses (Figures 2, 3, respectively).

### Running velocity

The average performance in the 800-m run was ∼156 ± 8 s, with individual times ranging from 138 to 164 s. This corresponds to an average running velocity of 17.6 ± 1.3 km.h^−1^. Following the initial 100 m (at a speed of 19.4 km/h), the running velocity gradually decreased. At the 300 m mark, it had reduced by −5.7 ± 4.6%, at the 500 m mark by −10.4 ± 8.3%, and it stabilized at the 700 m mark with a decline of −9.1 ± 13.5% compared to the initial velocity (all *P* < 0.001).

## Discussion and implications

### Summary of main findings

In an 800 m time trial where running velocity gradually decreased between 300 and 700 m, no changes in SA scores were detected at any distance intervals. This finding contradicts our initial hypothesis, as it suggests that alterations in gait asymmetries did not intensify as the run progressed. It is worth noting that this occurred despite the presence of fatigue-related changes in kinematics and kinetics, which were described elsewhere (3). Overall, the 800 m time trial did not result in increased mechanical constraints on one side of the body compared to the other.

During an 800 m time trial where running velocity progressively decreased from 300 to 700 m (3), no modification in SA scores was observed at any distance intervals. Contrary to our hypothesis, adjustments in gait asymmetries were not shown to be magnified throughout the run. This occurred despite the presence of fatigue-related kinematic and kinetic alterations, which were described elsewhere (3). Overall, running an 800 m time trial did not expose one side of the body to heightened mechanical constraints.

### Constant asymmetry throughout the run

The strong sense one gets from reading the Heil et al. (8) review is that studies assessing the biomechanical manifestation of fatigue on asymmetries have mixed findings, even for runs of the same distance (i.e., 10,000 m) with reports of unchanged (7) or increased side-to-side differences (10). In line with the well-preserved SA scores reported here for 800 m self-paced track running, two previous studies failed to measure meaningful asymmetry differences throughout a 5,000 m time trial [i.e., every 200 m (20)] and a constant-velocity 10,000 m treadmill run [i.e., at 1,500, 3,000, 5,000, 7,500 and 9,500 m distance intervals (7)]. Contrastingly, earlier research has documented that various biomechanical factors, such as knee internal rotation and knee stiffness (22), or anteroposterior accelerations (26), tend to exhibit increased asymmetry during fatigued running. Furthermore, SA scores for knee abduction moment, knee extension angles, and hip joint flexion moment have been shown to rise after participants completed running-induced fatigue protocols (9, 19). These seemingly discrepant findings concerning biomechanical manifestation of fatigue may be related to the diversity in the used protocols. For example, some studies use treadmill runs at constant velocity (7, 10), whereas other studies implement self-paced overground trials (20) to assess gait asymmetries during exhaustive runs. It is clear that the former methods are less appropriate for evaluating whether running-induced fatigue alters asymmetries because they could lead to an unnatural control of gait characteristics. Evaluating changes in bilateral leg asymmetry during self-paced runs, like 800 m track running, poses challenges in distinguishing true fatigue from auto-regulatory mechanisms. External regulation of running speed at 200 m intervals may help explore variations in symmetry, but its accuracy, especially during overground running, may be questionable.

Arguably, the adaptations from one side to the other are not solely mechanically driven but also depend on neuromuscular control strategies, which can be influenced by increasing fatigue, even when external load metrics (i.e., GRF signals) do not show asymmetry, as observed in this study. In a study by Jacques et al. (27), lower activation of the *soleus* muscle was observed in the dominant leg during the running phase of a simulated cycle-run transition, indicating a greater susceptibility to fatigue. Interestingly, such differences in muscle activation between legs were probably not caused by external loading since there were no kinetic and kinematic differences between the limbs. Future middle-distance investigations should therefore determine if a modification in inter-segmental coordination, that requires further EMG [i.e., timing and amplitude of muscle activation; Jacques et al. (27)] and kinematic [i.e., joint angles and moments (9)] data from dominant and non-dominant legs, would emerge to maintain constant asymmetries under fatigue.

### SA scores are inconsistent between vertically- vs. horizontally-derived variables

Another important finding is that, on average, vertically-derived measures exhibited the lowest degree of asymmetry when compared to mechanical variables calculated from the horizontal GRF signal. Similar observations have been made during single (15) or repeated treadmill sprints (11, 28) and a graded exercise test (29), although contradictory findings also exist [e.g., 30 m sprint on a non-motorized treadmill (30)]. A qualitative examination of the SA scores further suggests that asymmetries in peak forces and impulses (∼3%–8%) were approximately twice as large as those in phase durations (∼1%–2%). Consistent with previous research on perceptually-regulated interval running (31), adjustments occurring during the braking and push-off phases had comparable asymmetry scores. In contrast, deviations from symmetry, particularly for peak force, were reported to be two-to-three times larger during braking than during push-off phases in the context of repeated treadmill sprints (11) and across a range of constant velocities from low to high (6). Taken together, these observations emphasize that the extent of side-to-side differences depends on the specific metric, with variables derived from the horizontal GRF signal being the most asymmetrical.

### Individual responses

Consistent with earlier investigations (29, 31), the SA scores for most runners exhibited a range roughly double the magnitude of the mean value across all metrics. A plausible explanation for this phenomenon could be the substantial variation in running styles among participants, as we did not assess their foot strike patterns in this study (32). Other individual characteristics not measured in this study that can contribute to large inter-individual differences in gait asymmetry include leg length discrepancies, strength asymmetries, joint range of motion, and previous injury (33). Furthermore, a considerable proportion of our participant sample consisted of team sport players who often display asymmetry between their dominant and non-dominant sides, which is typically attributed to the repetitive and unilateral nature of certain sport-specific actions [i.e., exclusively using the dominant leg to kick the ball (33)]. It is crucial to differentiate between a broad trend derived from the group mean SA scores of all tested runners and the individual diversity in the asymmetry profile when quantifying bilateral leg differences during fatigued running. Depending on the “baseline” SA scores (i.e., at the 100 m mark), variations in the alterations of asymmetries during later distance intervals (i.e., 300–700 m intervals) may occur for individuals with higher or lower asymmetry values in “fresh” conditions (8). A suggestion for future studies is therefore to focus less on searching for universal fatigue-induced change in SA scores and more on exploring the emergence of context-specific compensatory strategies (34).

### Limitations and additional considerations

This study has several noteworthy limitations. Firstly, an important caveat was that due to the use of a 5 m force platform system, we analyzed only one stride (comprising one left and one right step) at each of the four distance intervals. Furthermore, we could not ascertain whether the initial ground contact was made with the left or right foot (or vice versa). Consequently, it can not be ruled out that the first step on the force plate may differ between laps for a given individual. We therefore relied on the SA score (24), a dimensionless measure of asymmetry that mitigates artificial inflation or problems associated with normalization to one of the two limbs, unlike other symmetry measures. In our context, this made the SA score a robust metric for assessing asymmetry when comparing kinetic variables. Secondly, the study's participants consisted of healthy, male physical education students who were not specialized in running. Consequently, it may not be appropriate to generalize our findings to athletes with a history of injury or differing running experience. Mo et al. (35) provided backing for this observation, noting that novice runners exhibited inconsistent asymmetries, recreational runners displayed their most symmetrical pattern at their preferred velocity, and competitive runners demonstrated a more symmetrical manner overall. Thirdly, it is important to note that a considerable portion of the total distance covered on a 200 m indoor track involves the bend portion. Although we collected GRFs at the end of the straight line, it can not be ruled out that SA scores (and fatigue-related asymmetries) might have shown variations if force data were collected on curved paths, introducing distinct mechanical constraints between the inside and outside legs (36). It is worth mentioning that gait asymmetry for contact time remained relatively consistent on both straight and curved sections (2.7% vs. 2.8%) during a 5,000 m time trial conducted on a 400 m synthetic track (20).

The absence of a significant time interval effect in our study may, in part, be attributed to the relatively modest sample size of eighteen participants. Although we did not conduct an *a priori* sample size calculation, it is worth mentioning that our sample size aligns with the range reported in a recent systematic review that explored the impact of exercise-induced fatigue on inter-limb asymmetries (8). Estimating an appropriate sample size for gait asymmetry studies poses challenges as it involves determining the main parameter of interest while considering expected power and effect size. As also emphasized in our study, relying on a single metric may not fully capture the comprehensive biomechanical response in asymmetry studies (33).

More sophisticated statistical approaches, such as statistical parametric mapping, are needed to quantify asymmetry in fatigued runners. These methods consider the entire gait cycle rather than discrete values (i.e., peak forces), and can determine if force traces differ between legs at specific periods during the gait cycle (27). To address potential variability in gait asymmetry, it may also be beneficial to consider using the symmetry function index (37) in future studies examining the entire gait cycle. This approach involves calculating the integral of the absolute difference between the left and right leg's stance phases, allowing for the analysis of symmetry from various perspectives, including sagittal, transverse, and frontal indices. To better understand the biomechanical manifestation of fatigue on side-to-side differences, it is recommended that future running studies include information about ankle, knee, and hip joint angles and angular velocities using a 3-D motion capture system. Indeed, previous studies assessing this aspect have reported increased bilateral asymmetries for gait kinematics (9, 19, 22). Finally, in our study, it was not verified whether the tested runners were rearfoot, midfoot, or forefoot strikers. Therefore, it remains unknown to what extent the habitual foot strike pattern influences the mechanical side-to-side differences during track running.

## Conclusion

In non-specialist runners, there were no discernible differences in asymmetries between vertically-derived parameters (i.e., contact time, peak vertical force, and vertical impulses) and horizontally-derived parameters (i.e., braking/push-off phase duration, peak braking/push-off forces, and braking/push-off impulses) during 800 m self-paced track running. Regardless of the distance interval, bilateral leg differences were approximately twice as large for the braking and push-off phases compared to vertical GRF asymmetry. While the mean SA scores provided in this study offer reference data, it is essential to note that these values should not serve as fixed benchmarks for expected asymmetry magnitude, as they are inherently influenced by individual characteristics and the specific task (33). Practically, experimental procedures for characterizing the gait pattern during 800-m track running could be simplified by collecting leg mechanical data from only one side.

## Data Availability

The raw data supporting the conclusions of this article will be made available by the authors, without undue reservation.
